# Autologous bone marrow stem cell transplantation via the hepatic artery for the treatment of hepatitis B virus-related cirrhosis: a PRISMA-compliant meta-analysis based on the Chinese population

**DOI:** 10.1186/s13287-020-01627-5

**Published:** 2020-03-05

**Authors:** Ani Sun, Wenni Gao, Ting Xiao

**Affiliations:** 1 Infection Control Office, Weifang People’s Hospital, Weifang, 261041 Shandong Province China; 2 Dispensing room for intravenous transfusion, Weifang People’s Hospital, Weifang, 261041 Shandong Province China; 3 Department of Infectious Diseases, Weifang People’s Hospital, Guangwen Street, No.151, Weifang, 261041 Shandong Province China

**Keywords:** Autologous bone marrow stem cell, Routine therapy, Hepatic artery, Hepatitis B virus-related cirrhosis, Meta-analysis

## Abstract

**Objective:**

Autologous bone marrow stem cell (ABMSC) transplantation has been considered a promising option for hepatitis B virus-related cirrhosis (HBV-C). Although an analysis of the published literature has been performed, the exact effects and safety have yet to be systematically investigated.

**Methods:**

We conducted a wide-ranging online search of electronic databases (Web of Science, PubMed, Cochrane Library, Embase, CNKI, VIP, and Wanfang database) to reach systematic conclusions. Outcome measurements, including therapeutic efficacy, clinical symptoms, and adverse events, were extracted and analyzed statistically.

**Results:**

Ultimately, a total of 10 articles including 662 HBV-C patients were included in this analysis, which indicated that ABMSC therapy could significantly improve liver function in patients with HBV-C in terms of the MELD and Child-Pugh scores, total bilirubin, serum albumin, alanine aminotransferase, aspartate aminotransferase, and coagulation function. Compared with patients receiving routine therapy (RT), those treated with ABMSC and RT combined therapy showed improved clinical symptoms, as represented by increased appetite and reduced fatigue and ascitic fluid and abdominal distension. Moreover, the fibrosis indexes indicated a reduction in liver fibrosis in patients treated with combined therapy according to the improved levels of hyaluronic acid (MD = − 70.47, CI = − 103.72–37.21, *P* <  0.0001), laminin (MD = − 25.11, CI = − 37.73–12.49, *P* <  0.0001), type III procollagen (MD = − 22.42, CI = − 34.49–10.34, *P* = 0.0003), and type IV collagen (MD = − 22.50, CI = − 39.92–5.08, *P* = 0.01). No obvious adverse events occurred during ABMSC treatment.

**Conclusion:**

ABMSC transplantation via the hepatic artery was safe and effective in treating HBV-C without causing severe adverse events.

## Introduction

There are currently over 350 million people with chronic hepatitis B (CHB) worldwide, and hepatitis B virus (HBV) infection is one of the main causes of liver cirrhosis (LC) [1, 2]. HBV is the tenth leading cause of death. More than one million HBV carriers die of LC and liver cancer every year [1]. As reported by the World Health Organization, 45% of the population lives in high-prevalence CHB areas [3]. CHB is particularly prevalent in China, where 7.18% of the population aged 1 to 59 years is a chronic HBV surface antigen carrier [4–6]. Among untreated CHB patients, 6–20% develop to cirrhosis in 5 years [4, 7]. Untreated decompensated cirrhosis patients show a poor prognosis, with a 5-year-survival rate ranging from 14 to 35% [4, 7].

Currently, clinical treatments for CHB include interferon-α (IFN-α) injection and orally administered nucleotide analogs (NAs), such as adefovir dipivoxil, entecavir, lamivudine, tenofovir, and telbivudine [8]. As NAs suppress HBV replication only at the point of DNA synthesis progression, most patients require long-term treatment, which is unfortunately limited by drug resistance [9]. Although IFN-α has both antiviral and immunomodulatory properties against HBV, it performs poorly in suppressing HBV DNA replication [8]. In addition, its clinical application is very limited by contraindications, such as hematological and neurological diseases. It is also not applicable for decompensated hepatitis B virus-related cirrhosis (HBV-C), as it may lead to liver failure [8, 10]. Therefore, the development of an effective therapeutic method is needed.

The rapid development of stem cell research has been gaining attention for cirrhosis therapy, as transplanted stem cells have been reported to be beneficial for cirrhosis [11–15]. Autologous bone marrow stem cell (ABMSC) contains several types of stem cells, including mesenchymal stem cells, hepatic progenitor cells, and hematopoietic stem cells [16]. These multipotent stem cells can migrate to lesion sites, differentiate into hepatocytes, and secrete various cytokines and growth factors [17, 18]. This mechanism suggests that ABMSC transplantation may be a potential treatment strategy for cirrhosis.

Several clinical trials have reported that ABMSC transfusion alleviates liver fibrosis and improves liver functions without causing severe side effects [2, 19, 20]. Preclinical studies of ABMSC transplantation, particularly for HBV-C, have also been conducted, but they have utilized various individual therapeutic regimens. In this study, we focused on ABMSC transplantation performed specifically via the hepatic artery when conducting a meta-analysis of published clinical trials to provide a scientific reference for upcoming clinical research and future applications.

## Material and methods

This meta-analysis was performed in accordance with the Preferred Reporting Items for Systematic Reviews and Meta-Analyses (PRISMA) guidelines. Ethical approval was not necessary because this study was a meta-analysis.

### Data sources and selection criteria

The analyzed literature was searched across the Web of Science, PubMed, Cochrane Library, Embase, China National Knowledge Infrastructure (CNKI), Chinese Scientific Journal Database (VIP), and Wanfang database by May 2018. The search was performed with the following key terms: “stem cells” or “bone marrow stem cells” or “mesenchymal stem cells” or “bone marrow mesenchymal stem cells” AND “cirrhosis” or “liver cirrhosis” or “viral cirrhosis” or “hepatitis B virus-related cirrhosis”.

The retrieved literature was reviewed, and those meeting the following inclusion criteria were involved in this study: (1) case-controlled clinical trials, (2) patients with HBV-C, (3) patients who had no hepatocellular carcinoma or other malignant tumor and who were without pregnancy or lactation, (4) patients in the experimental group who received ABMSC transplantation and routine therapy (RT) combined therapy and patients in the control group who were treated with RT alone, and (5) patients treated with ABMSC transfusion who received treatment via the hepatic artery.

### Data extraction and quality assessment

Literature screening and data extraction were carried out by two independent authors (Ani Sun and Wenni Gao) and verified by a third reviewer (Ting Xiao). All included studies were summarized as follows: first authors’ names, year of publication, HBV-C stages, sample sizes, therapeutic regimens, administration route, dosages of ABMSC, enrollment period, follow-up duration, and evaluation parameters. The methodological quality of the included studies was assessed according to the Cochrane Handbook [21].

### Outcome definition

The outcomes of greatest interest included treatment efficacy, clinical symptoms, and adverse events. Treatment efficacy was assessed in terms of total bilirubin (TBIL), serum albumin (ALB), alanine aminotransferase (ALT), and aspartate aminotransferase (AST) levels, prothrombin time (PT), prothrombin activity (PTA), model for end-stage liver disease (MELD), and Child-Pugh score, and liver fibrosis indexes included hyaluronic acid (HA), laminin (LN), type III procollagen (PC III), and type IV collagen (CIV) levels. Clinical symptoms of patients were also evaluated based on fatigue, appetite, ascetics, and abdominal distension. Adverse events that occurred during therapy were also considered in the assessment.

### Statistical analysis

We performed a comparative analysis between patients treated with RT alone and those treated with ABMSC transfusion and RT combined therapy with Review Manager 5.3 (Cochrane Collaboration) and Stata 13.0 (Stata Corporation). *P* <  0.05 indicated a statistically significant difference. Cochran’s *Q* test was conducted to assess the heterogeneity cross the involved studies, and *I*^*2*^ < 50% or *P* > 0.1 indicated the studies were homogenous [22]. A fixed effects model was used to pool the estimates when heterogeneity was absent. Otherwise, a random effects model was selected. Dichotomous data were represented by the odds ratio (OR) with the respective 95% confidence interval (CI), whereas continuous variables were expressed as mean difference (MD) with 95% CI. Publication bias was evaluated based on the funnel plot and Begg’s and Egger’s tests. Sensitivity analyses were also performed to assess the impact of cell dosages and the sample sizes of the involved studies.

## Results

### Search results

**Fig. 1:**
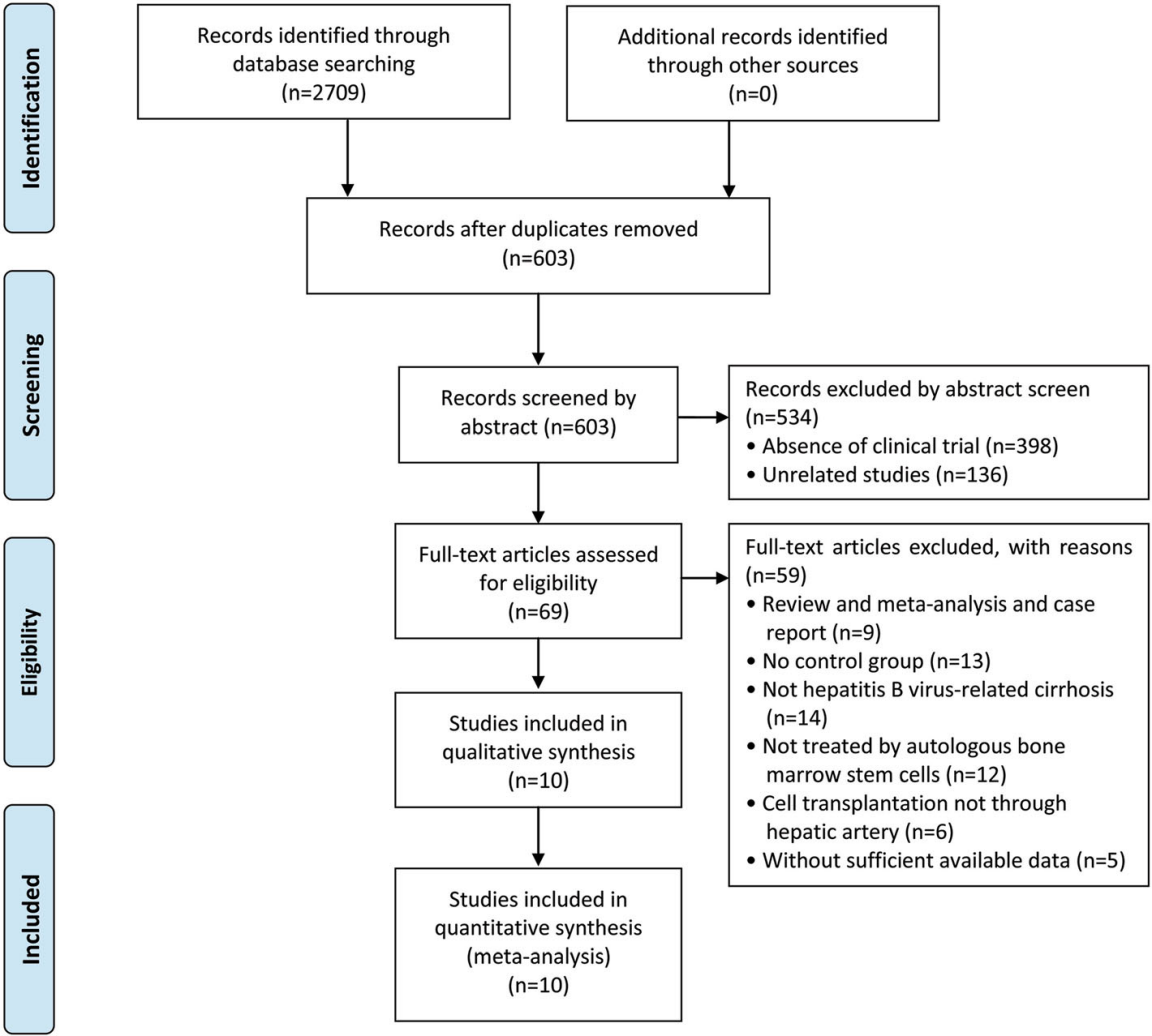
Flow diagram of the selection process

Of 2709 articles that were preliminarily screened for the initial review, 2106 were excluded due to duplication. After the title and abstract review, 534 articles were further excluded due to the lack of clinical trials (*n* = 398) and unrelated studies (*n* = 136), resulting in 69 potentially relevant studies. After a detailed assessment of the full texts, articles designated as reviews and meta-analyses and case reports (*n* = 9), studies without a control group (*n* = 13), trials unrelated to HBV-C (*n* = 14) and ABMSC therapy (*n* = 12), ABMSC transplantation not via hepatic artery infusion (*n* = 6), and papers with insufficient data (*n* = 5) were excluded. Finally, 10 studies [2, 19, 20, 23–29] including 622 HBV-C patients met the inclusion criteria for our meta-analysis (Fig. 1).

### Patient characteristics

All included trials that met the inclusion criteria were conducted in China and included 286 HBV-C patients treated with hepatic artery-administered ABMSC and RT combined therapy and 336 patients treated with RT alone. Among all the included studies, 9 studies [2, 19, 23–29] included patients with decompensated cirrhosis, and the remaining study [20] lacked a clear description of the stages of HBV-C. Detailed information regarding the involved studies and participants is shown in Table 1.

**Table 1:** Information of ABMSC therapy

Included studies	Stage (Child-Pugh)	Patients (Con/Exp)	Therapeutic regimen (administration route)	Cell dose	Enrollment period	Follow-up (week)	Parameter types
Chen et al. [23]	B–C	34/33	RT+ABMSC (HA)	3.0–5.0 × 10^6^	November 2008–April 2012	24	TBIL, ALB, ALT, PT, PTA, MELD, Child-Pugh
Cui [24]	B–C	22/18	RT+ABMSC (HA)	5.6 × 10^8^–1.8 × 10^11^	October 2011–December 2013	12	TBIL, ALB, ALT, PTA, CS, LFI, MELD, Child-Pugh
Hou et al. [25]	ND	25/25	RT+ABMSC (HA)	ND	November 2009–February 2011	16	TBIL, ALB, ALT, PT, MELD
Jiang [26]	B–C	13/12	RT+ABMSC (HA)	2.1–6.8 × 10^10^	July 2009–June 2011	24	TBIL, ALB, ALT, AST, PT, CS
Jin et al. [27]	B–C	20/20	RT+ABMSC (HA)	4.0 × 10^7^–3.0 × 10^8^	April 2009–April 2010	12	TBIL, ALB, PTA
Liu et al. [19]	B–C	37/40	RT+ABMSC (HA)	3.2 × 10^10^–1.6 × 10^11^	April 2009–October 2010	4	TBIL, ALB, ALT, AST, PT
Mao et al. [28]	A–C	32/32	RT+ABMSC (HA)	> 10^9^	January 2009–January 2012	4	TBIL, ALB, ALT, PT
Peng et al. [2]	ND	77/39	RT+ABMSC (HA)	3.4 ± 3.8 × 10^8^	May 2005–June 2009	4	TBIL, ALB, ALT, PT, MELD
Wu et al. [29]	B–C	25/27	RT+ABMSC (HA)	1.0 × 10^8–9^	January 2013–January 2015	24	TBIL, ALT, AST, CS, LFI, MELD, Child-Pugh
Xu et al. [20]	ND	29/27	RT+ABMSCs (HA)	8.5 ± 3.3 × 10^8^	March 2012–December 2012	24	ALB, ALT, MELD

*Abbreviations*: *Con* control group (RT alone group), *Exp* experimental group (RT plus ABMSC therapy), *RT* routing therapy, *ABMSC* autologous bone marrow stem cell, *ND* non-determined, *HA* hepatic artery

### Quality assessment

**Fig. 2:**
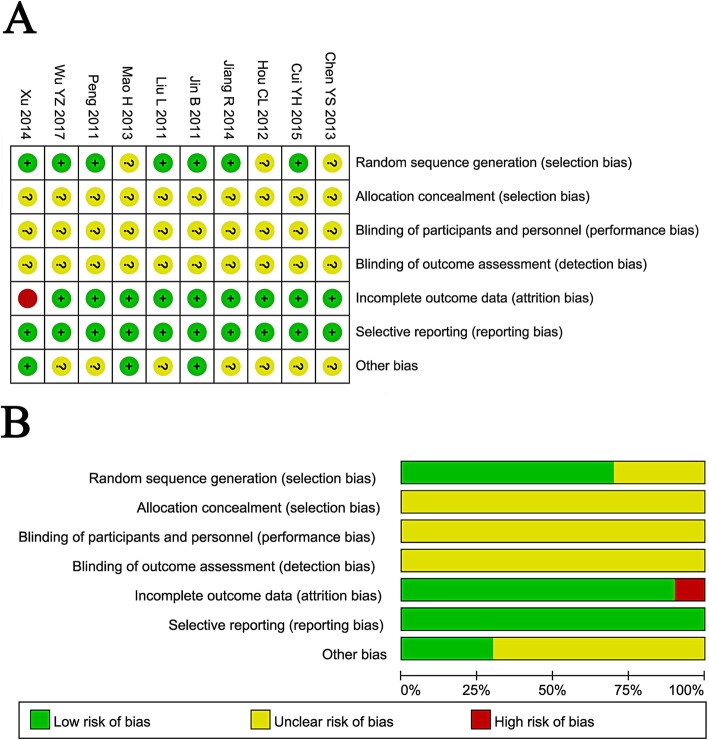
Risk of bias summary: review of authors’ judgments about each risk of bias item for included studies (**a**). Risk of bias graph: review of authors’ judgments about each risk of bias item presented as percentages across all included studies (**b**). Note: Each color represents a different level of bias: red for high-risk, green for low-risk, and yellow for unclear risk of bias

The assessment of bias risk is shown in Fig. 2. Seven of the 10 involved trials were determined as having a low risk of bias, while the other 3 trials did not provide a clear description of the randomization process. All trials provided a clear description of the selection, performance, and detection and hence were designated as having an unclear risk of bias. One study missing follow-up study was regarded as having a high risk of attrition bias. All 10 studies were free of reporting risks.

### Therapeutic efficacy assessments

#### Evaluation of biochemical indexes (TBIL, ALB, ALT, and AST)

HBV-C patient status can be reflected by multiple markers, such as TBIL, ALB, ALT, AST, PT, and PTA. Before treatment, no obvious differences were observed in these indicators between the experimental and control groups (Supplementary Figure 1).

**Fig. 3:**
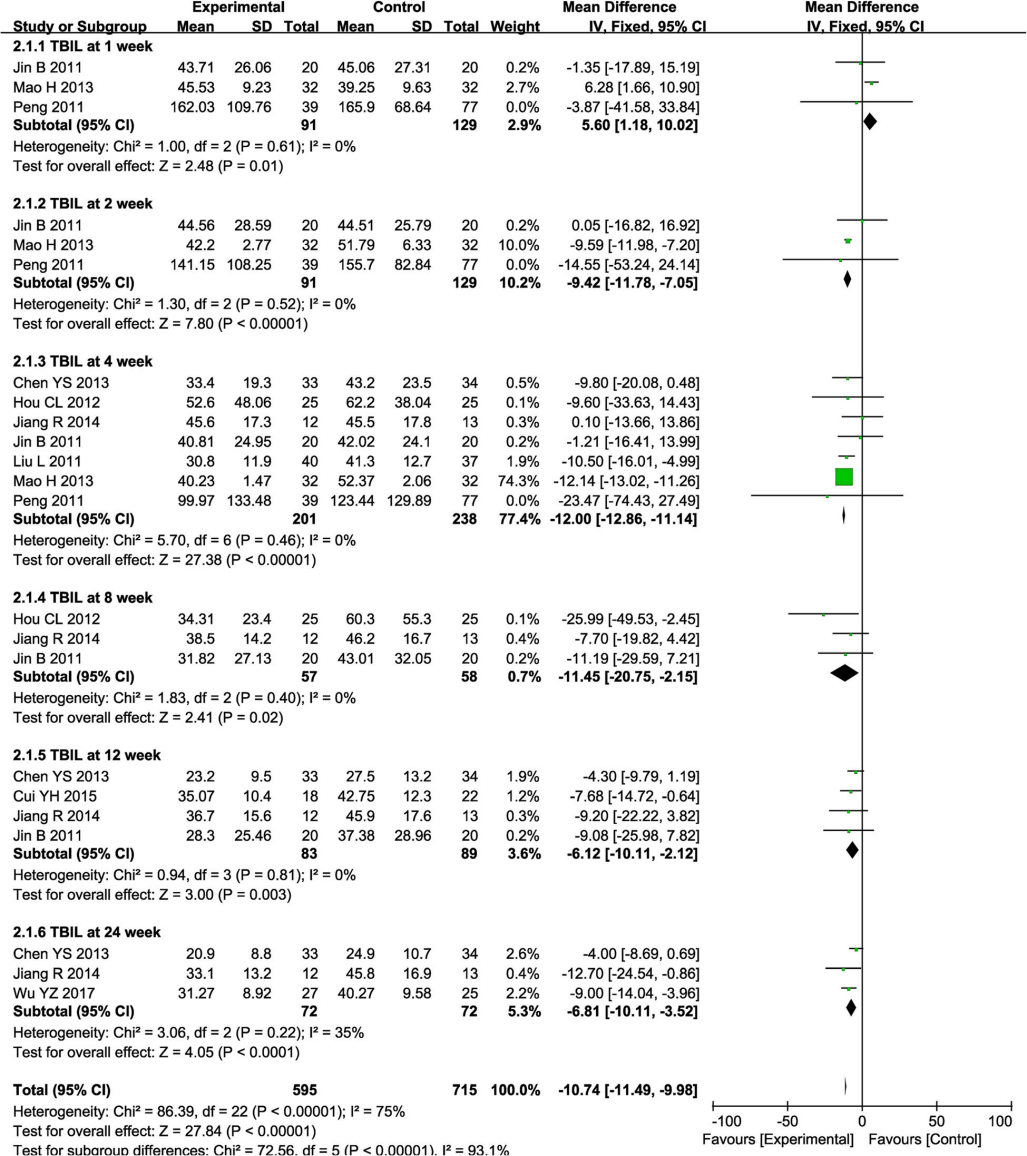
Forest plot of the comparison of total bilirubin (TBIL) between the experimental and control group. Control group, RT alone group; experimental group, RT plus ABMSC therapy; ABMSC, autologous bone marrow stem cell; RT, routing therapy. The fixed-effects meta-analysis model (inverse variance method) was used

After treatment, the TBIL level was significantly lower in the combined group at weeks 2, 4, 8, 12, and 24 compared with RT alone (Fig. 3, 2nd: MD = − 9.42, CI = − 11.78–7.05, *P* < 0.00001; 4th: MD = − 12.00, CI = − 12.86–11.14, *P* < 0.00001; 8th: MD = − 11.45, CI = − 20.75–2.15, *P* = 0.02; 12th: MD = − 6.12, CI = − 10.11–2.12, *P* = 0.003; 24th: MD = − 6.81, CI = − 10.11–3.52, *P* <  0.0001). Most studies reported a decreased TBIL level after combined therapy, except the study by Mao et al. showing an increased TBIL level at week 1 after ABMSC therapy, which may require further discussion.

**Fig. 4:**
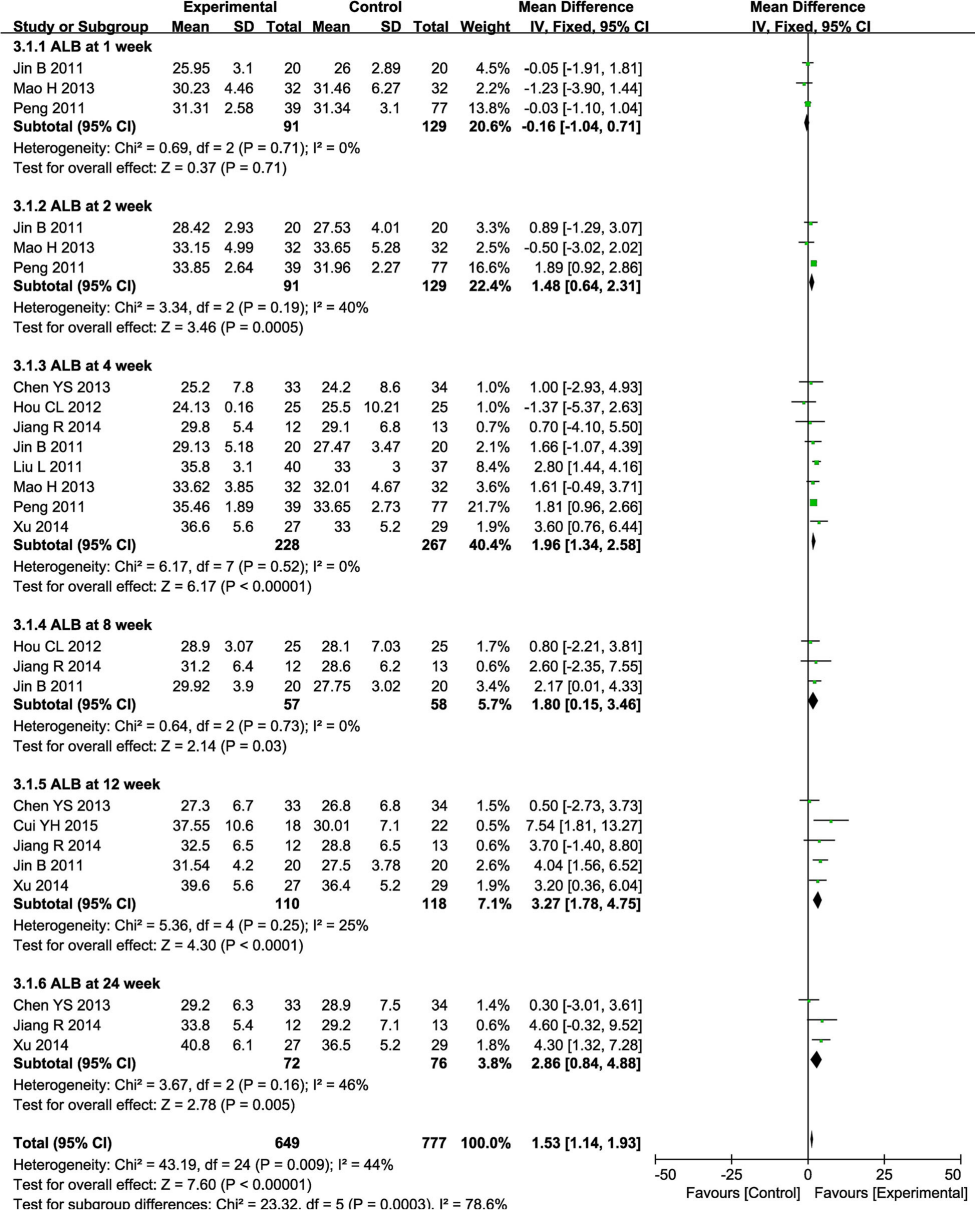
Forest plot of the comparison of albumin (ALB) between the experimental and control group. Control group, RT alone group; experimental group, RT plus ABMSC therapy; ABMSC, autologous bone marrow stem cell; RT, routing therapy. The fixed-effects meta-analysis model (inverse variance method) was used

The ALB level was significantly higher in the combined therapy group than the control group at weeks 2, 4, 8, 12, and 24 after therapy (Fig. 4, 2nd: MD = 1.48, CI = 0.64–2.31, *P* = 0.0005; 4th: MD = 1.96, CI = 1.34–2.58, *P* <  0.00001; 8th: MD = 1.80, CI = 0.15–3.46, *P* = 0.03; 12th: MD = 3.27, CI = 1.78–4.75, *P* <  0.0001; 24th: MD = 2.86, CI = 0.84–4.88, *P* = 0.005).

**Fig. 5:**
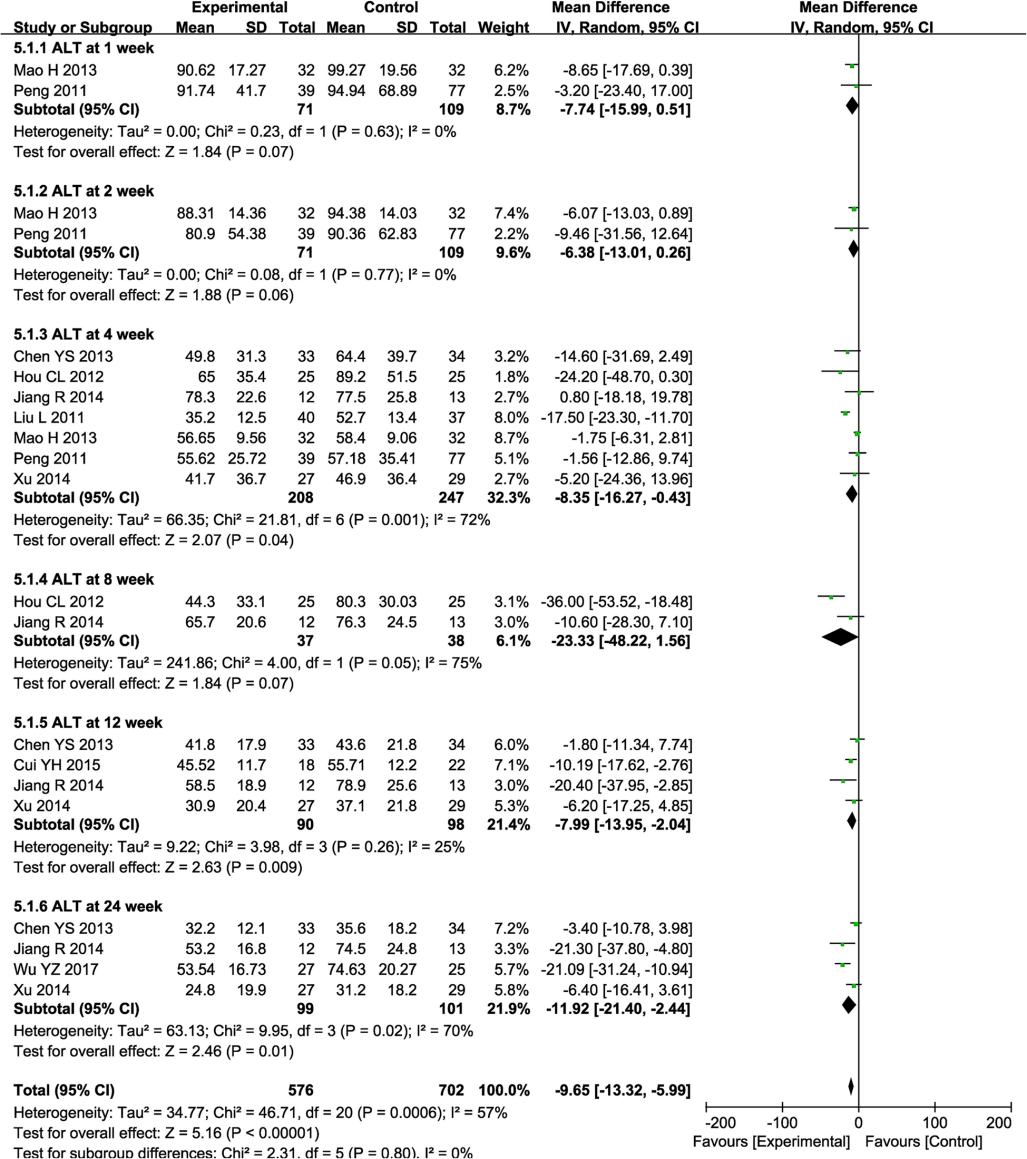
Forest plot of the comparison of alanine aminotransferase (ALT) between the experimental and control group. Control group, RT alone group; experimental group, RT plus ABMSC therapy; ABMSC, autologous bone marrow stem cell; RT, routing therapy. The random effects meta-analysis model (inverse variance method) was used

The ALT level was significantly decreased after combined treatment, especially at weeks 4, 12, and 24 (Fig. 5, 4th: MD = − 8.35, CI = − 16.27–0.43, *P* = 0.04; 12th: MD = − 7.99, CI = − 13.95–2.04, *P* = 0.009; 24th: MD = − 11.92, CI = − 21.40–2.44, *P* = 0.01), while it was insignificantly reduced at weeks 1, 2, and 8 (Fig. 5, 1st: MD = − 7.74, CI = − 15.99–0.51, *P* = 0.07; 2nd: MD = − 6.38, CI = − 13.01–0.26, *P* = 0.06; 8th: MD = − 23.33, CI = − 48.22–1.56, *P* = 0.07).

**Fig. 6:**
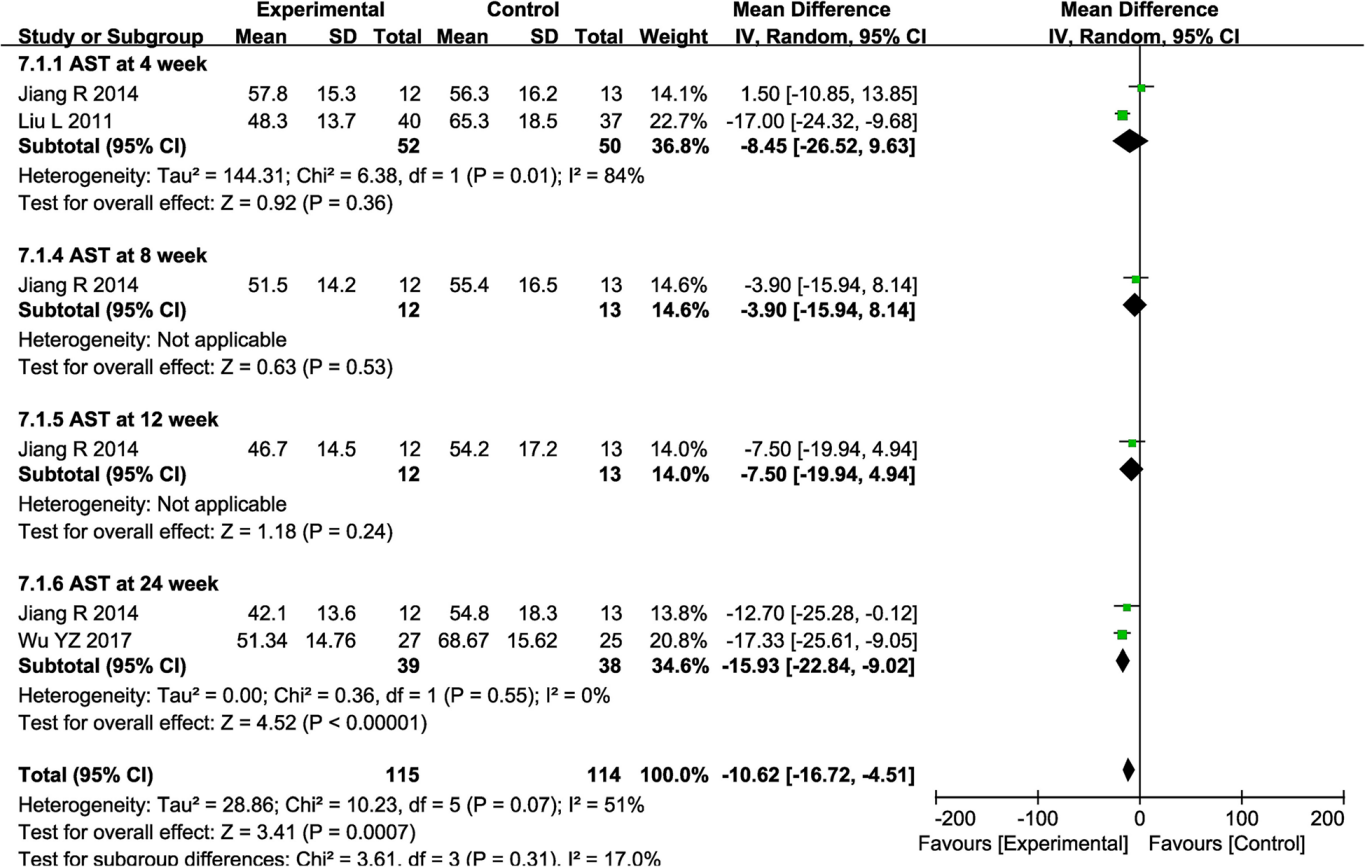
Forest plot of the comparison of aspartate aminotransferase (AST) between the experimental and control group. Control group, RT alone group; experimental group, RT plus ABMSC therapy; ABMSC, autologous bone marrow stem cell; RT, routing therapy. The random effects meta-analysis model (inverse variance method) was used

The AST level was significantly decreased after combined treatment only at week 24 (Fig. 6, 4th: MD = − 8.45, CI = − 26.52-9.63, *P* = 0.36; 8th: MD = − 3.90, CI = − 15.94–8.14, *P* = 0.53; 12th: MD = − 7.50, CI = − 19.94–4.94, *P* = 0.24; 24th: MD = − 15.93, CI = − 22.84–9.02, *P* <  0.00001).

#### Evaluation of coagulation function

**Fig. 7:**
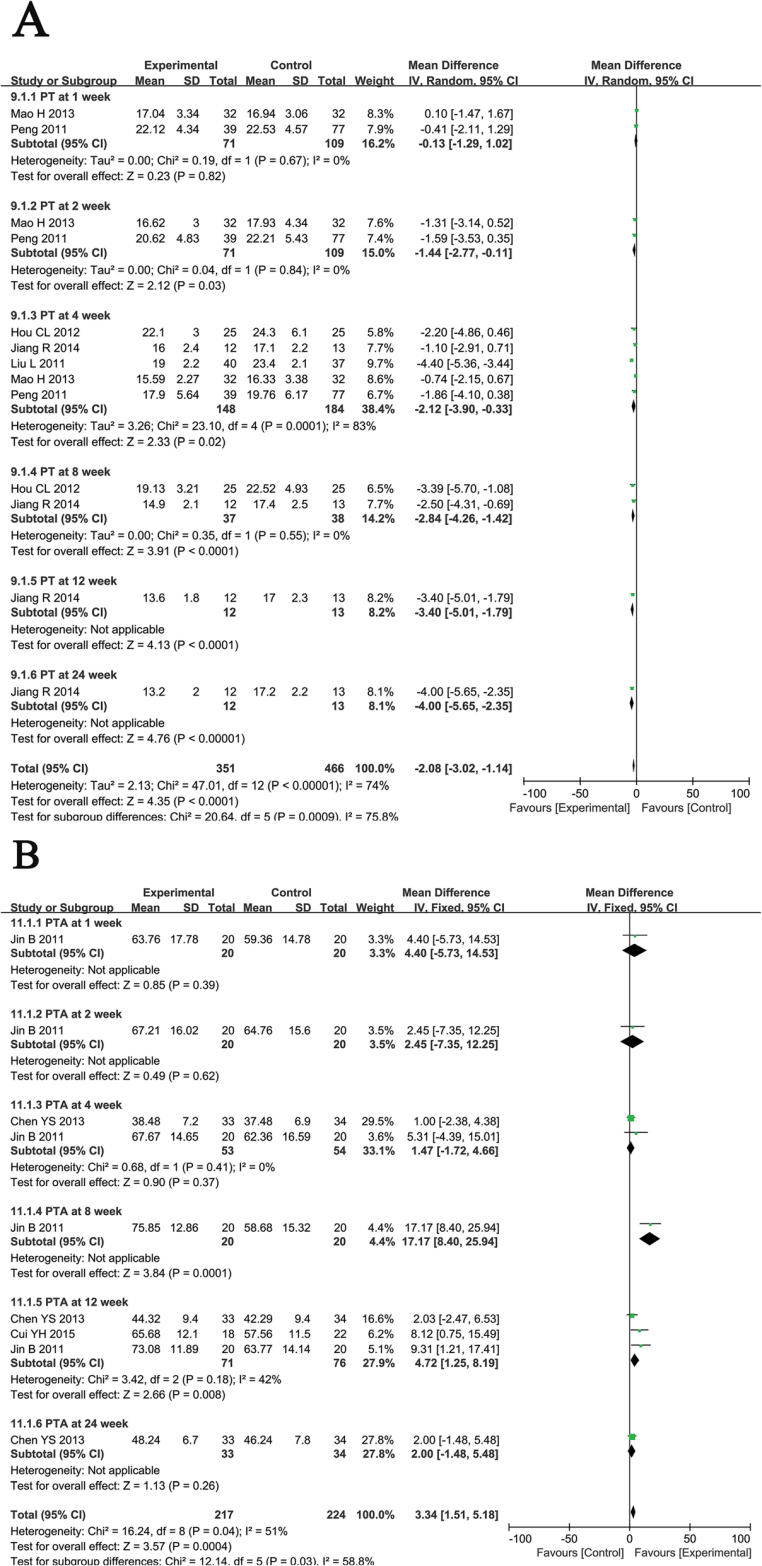
Forest plot of the comparison of coagulation function including prothrombin time (PT, **a**) and prothrombin activity (PTA, **b**) between the experimental and control group. Control group, RT alone group; experimental group, RT plus ABMSC therapy; ABMSC, autologous bone marrow stem cell; RT, routing therapy

PT and PTA are important indicators of coagulation function in patients. Patients who received the combination therapy had significantly greater reductions in PT than those treated with RT at weeks 2, 4, 8, 12, and 24 (Fig. 7a, 2nd: MD = − 1.44, CI = − 2.77–0.11, *P* = 0.03; 4th: MD = − 2.12, CI = − 3.90–0.33, *P* = 0.02; 8th: MD = − 2.84, CI = − 4.26–1.42, *P* <  0.0001; 12th: MD = − 3.40, CI = − 5.10–1.79, *P* < 0.0001; 24th: MD = − 4.00, CI = − 5.65–2.35, *P* < 0.00001). Moreover, the combined treatment group experienced a significantly greater increase in PTA than the control group at weeks 8 and 12 (Fig. 7b, 8th: MD = 17.17, CI = 8.40–25.94, *P* = 0.0001; 12th: MD = 4.72, CI = 1.25–8.19, *P* = 0.008).

#### Evaluation of hepatic fibrosis indicators

**Fig. 8:**
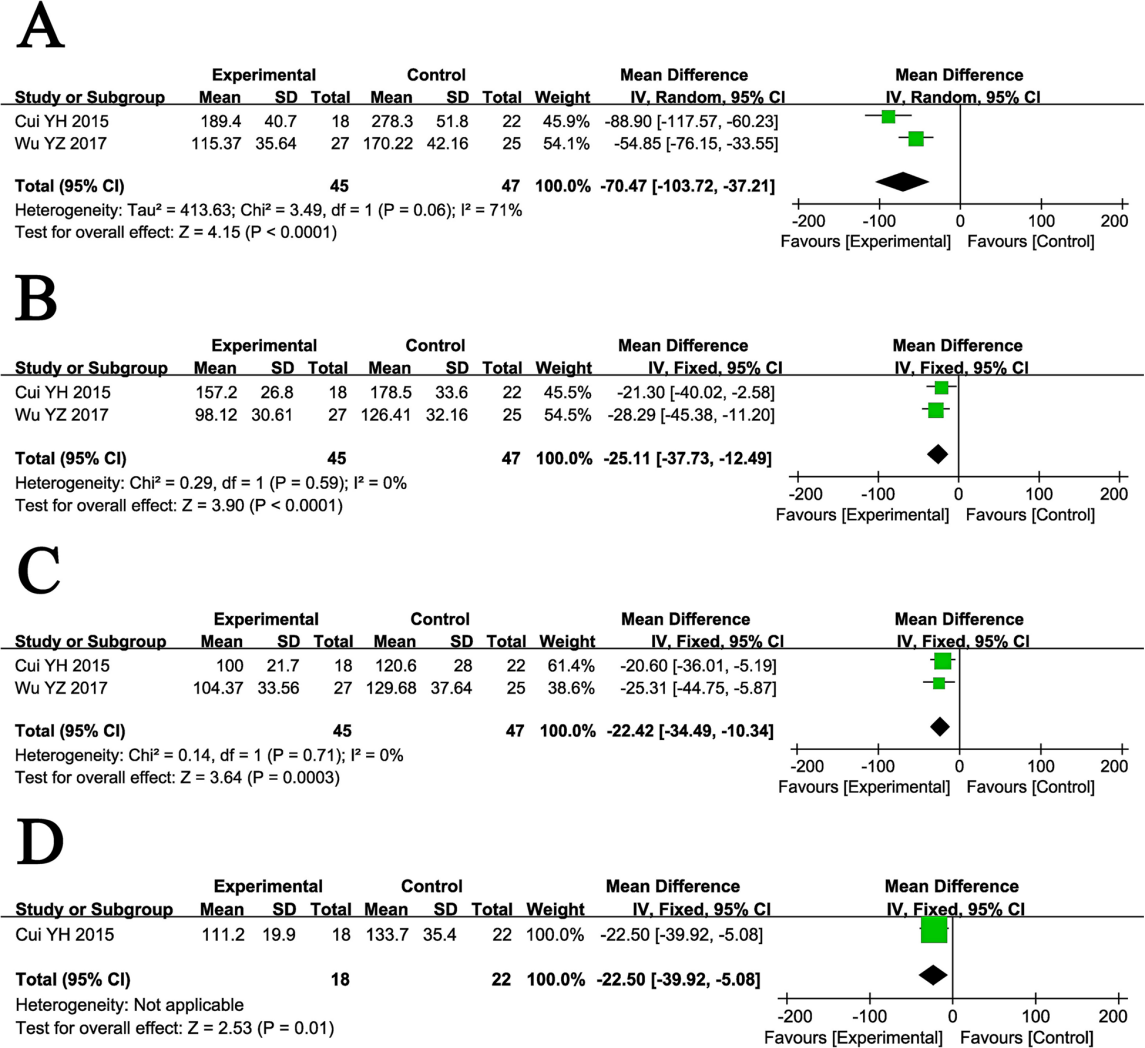
Forest plot of the comparison of serum liver fibrosis markers including hyaluronic acid (HA, **a**), laminin (LN, **b**), type III procollagen (PC III, **c**), and type IV collagen (CIV, **d**) between the experimental and control group. Control group, RT alone group; experimental group, RT plus ABMSC therapy; ABMSC, autologous bone marrow stem cell, RT, routing therapy

Serum fibrosis indicators were analyzed, including HA, LN, PC III, and CIV. Before treatment, these fibrosis markers did not show differences between the two groups (Supplementary Figure 2). After treatment, the levels of HA, LN, PC III, and CIV in patients who received combined therapy were all significantly improved compared with those treated with RT alone (Fig. 8, HA: MD = − 70.47, CI = − 103.72–37.21, *P* < 0.0001; LN: MD = − 25.11, CI = − 37.73–12.49, *P* < 0.0001; PC III: MD = − 22.42, CI = − 34.49–10.34, *P* = 0.0003; CIV: MD = − 22.50, CI = − 39.92–5.08, *P* = 0.01).

#### MELD and Child-Pugh scores

**Fig. 9:**
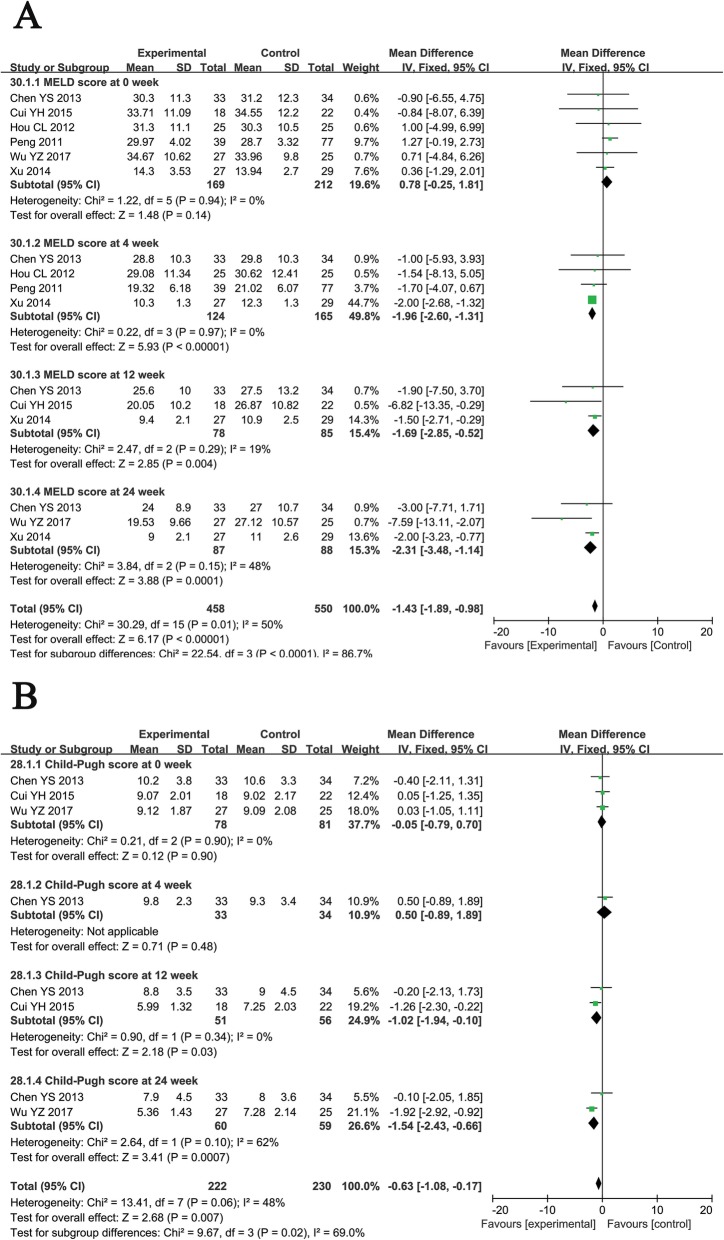
Forest plot of the comparison of model for end-stage liver disease (MELD, **a**) and Child-Pugh score (**b**) between the experimental and control group. Control group, RT alone group; experimental group, RT plus ABMSC therapy; ABMSC, autologous bone marrow stem cell; RT, routing therapy. The fixed-effects meta-analysis model (inverse variance method) was used

Before treatment, no differences were observed in the MELD and Child-Pugh scores between the two groups. Compared with patients treated with RT alone, ABMSC therapy was associated with a significantly lower MELD score at weeks 4, 12, and 24 (Fig. 9a, 4th: MD = − 1.96, CI = − 2.60–1.31, *P* < 0.00001; 12th: MD = − 1.69, CI = − 2.85–0.52, *P* = 0.004; 24th: MD = − 2.31, CI = − 3.48–1.14, *P* = 0.0001), and Child-Pugh score at weeks 12 and 24 (Fig. 9b, 12th: MD = − 1.02, CI = − 1.94–0.10, *P* = 0.03; 24th: MD = − 1.54, CI = − 2.43–0.66, *P* = 0.0007), indicating a more favorable prognosis.

#### Clinical symptom assessment

**Fig. 10:**
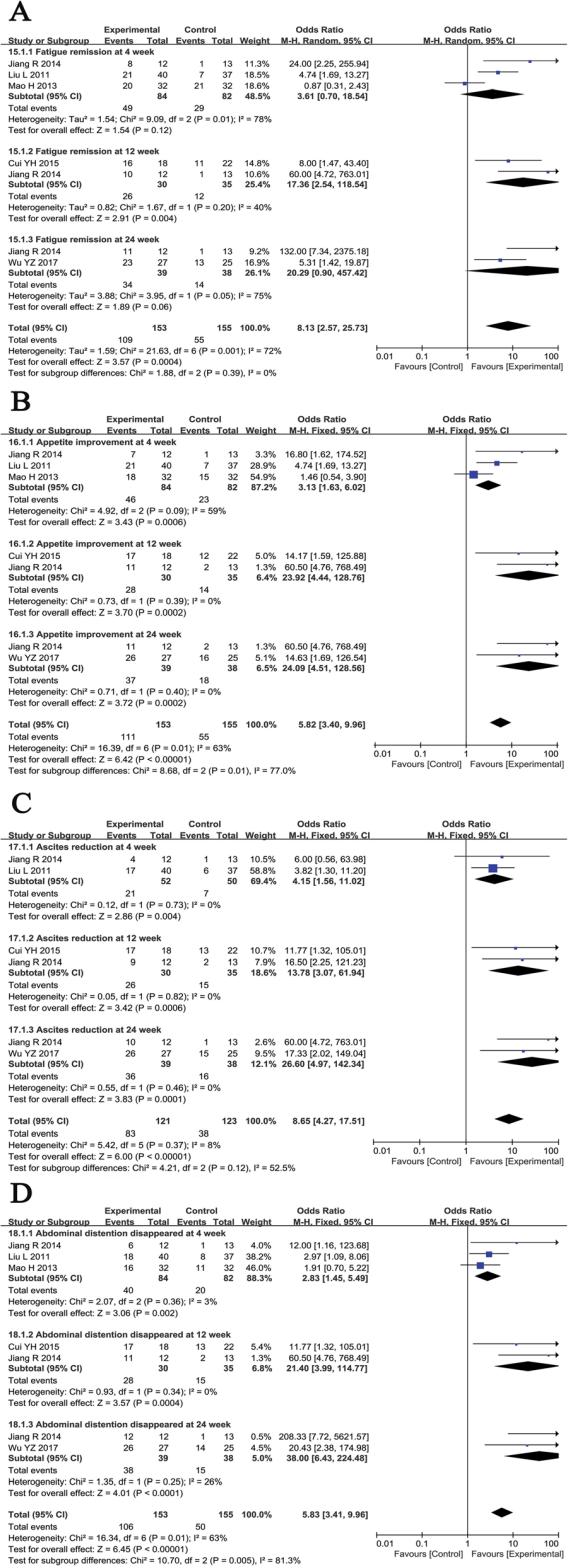
Forest plot of the comparison of clinical symptoms of patients including fatigue (**a**), appetite (**b**), abdominal distension (**c**), and ascetics (**d**) between the experimental and control group. Control group, RT alone group; experimental group, RT plus ABMSC therapy; ABMSC, autologous bone marrow stem cell; RT, routing therapy

The clinical symptoms of patients who received combined therapy were significantly improved compared with those of patients treated with RT alone (Fig. 10), as indicated by improved fatigue at week 12 (OR = 17.36, CI = 2.54–118.54, *P* = 0.004) and improved appetite (4th: OR = 3.13, CI = 1.63–6.02, *P* = 0.0006; 12th: OR = − 23.92, CI = 4.44–128.76, *P* = 0.0002; 24th: OR = 24.09, CI = 4.51–128.56, *P* = 0.0002), reduced ascitic fluid (4th: OR = 4.15, CI = 1.56–11.02, *P* = 0.004; 12th: OR = 13.78, CI = 3.07–61.94, *P* = 0.0006; 24th: OR = 26.60, CI = 4.97–142.34, *P* = 0.0001), and abdominal distension (4th: OR = 2.83, CI = 1.45–5.49, *P* = 0.002; 12th: OR = 21.40, CI = 3.99–114.77, *P* = 0.0004; 24th: OR = 38.00, CI = 6.43–224.48, *P* < 0.0001) at weeks 4, 12, and 24.

#### Adverse event assessment

Safety was evaluated upon assessing the adverse effects that occurred during and after treatment. The most common side effects during treatment were fever, nausea, and vomiting, and they usually subsided within 24 h without treatment. However, none of the trials compared the incidence of side effects between the experimental and control groups (Table 2).

**Table 2:** Information of adverse effects during the ABMSC therapy

Included studies	Adverse events (number)
Chen et al. [23]	Fever (2), nausea (8), ecchymosis (16)
Cui [24]	Nausea and vomiting (5), fever (3)
Hou et al. [25]	Low-grade fever (2), digestive tract hemorrhage (1)
Jiang [26]	Low-grade fever (1), pain (1), blood oozing from the wound (1)
Jin et al. [27]	No obvious adverse reactions
Liu et al. [19]	Low-grade fever (1), nausea (2), blood oozing from the wound (1)
Mao et al. [28]	No obvious adverse reactions
Peng et al. [2]	No obvious adverse reactions
Wu et al. [29]	Nausea and vomiting (4), fever (2), diarrhea (1)
Xu et al. [20]	Low-grade fever (1)

*Abbreviations*: *ABMSC* autologous bone marrow stem cell, *ND* non-determined

#### Publication bias

**Fig. 11:**
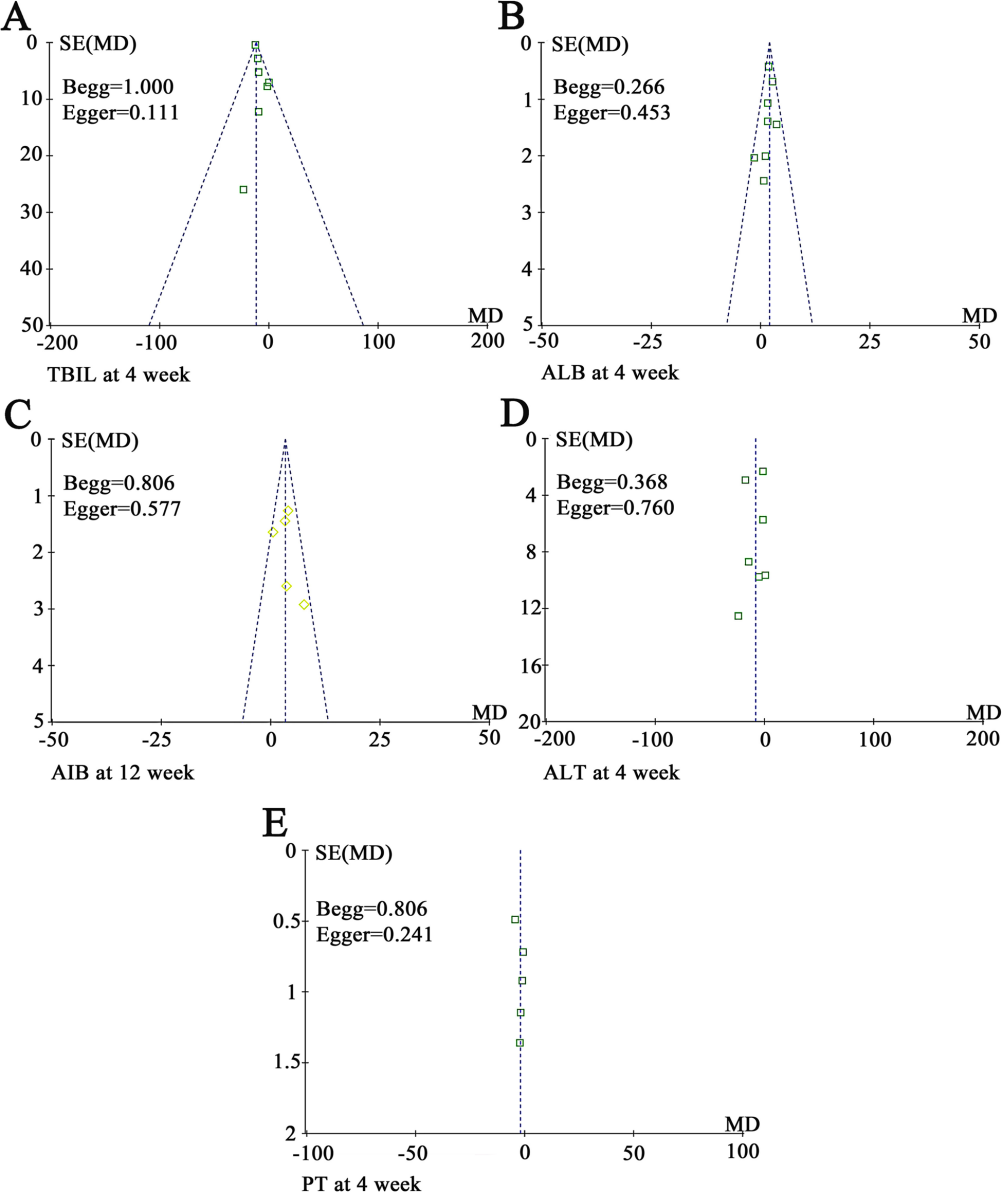
Funnel plot of percentage of total bilirubin (TBIL, **a**), albumin (ALB, **b** and **c**), alanine aminotransferase (ALT, **d**), and prothrombin time (PT, **e**). Notes: Parameters discussed in over 5 papers were conducted bias analyses

Funnel plots drawn for the studies on primary outcomes (TBIL, ALB, ALT, and PT) were approximately symmetrical, indicating the adequate control of publication bias and the reliability of our primary conclusions (Fig. 11). We further assessed publication bias by Begg’s and Egger’s regression tests, and the results were consistent with funnel plots.

#### Sensitivity analysis

A sensitivity analysis was conducted, and one trial [20] was excluded because it lacked a clear description of the stages of HBV-C (compensatory or decompensatory stage). The results of this analysis were similar to those obtained from the overall analysis of the pooled trials (Supplementary Figures 3, 4, and 5).

Moreover, we conducted a subgroup analysis to explore the source of heterogeneity in TBIL, ALB, ALT, and PT with respect to the dosage of injected cells and sample size of the study {According to the estimation formula of sample size [*n* = (Uα + Uβ)^2^ 2P(1 − P)/(P1 − P0)^2^; *α* = 0.05, *β* = 0.10] [30], a sample size greater than 50 is appropriate to evaluate the efficacy of ABMSC for HBV-C. Therefore, we conducted a subgroup analysis according to the different sample size (study sample size > 50 or ≤ 50) in our study. In other words, the results of clinical trials with a sample size greater than 50 are more reliable than small-scale clinical trials (study sample size ≤ 50)}. As shown in Table 3, our results showed that stem cell therapy was more effective with a higher cell dose (cell number > 1 × 10^10^) and large sample size (study sample size > 50), as indicated by decreased TBIL and increased ALB.

**Table 3:** Subgroup analyses of TBIL, ALB, ALT, and PT between the experimental and control group at the 4th week after therapy

Parameter	Factors at study level	Exp group	Con group	Analysis method	Heterogeneity		Mean difference (MD)	95% CI	*P* value
		No. patients (*n*)	No. patients (*n*)		*I*^2^ (%)	*P* value			
TBIL	**Cell number**								
	> 1 × 10^10^	52	50	Fixed	49	0.16	− 9.04	− 14.15 to − 3.92	0.0005
	< 1 × 10^10^	59	97	Fixed	0	0.41	− 3.03	− 17.60 to 11.54	0.68
	**Study sample size**								
	> 50	144	180	Fixed	0	0.87	− 12.09	− 12.95 to − 11.12	< 0.00001
	≤ 50	57	58	Fixed	0	0.79	− 1.88	− 11.27 to 7.51	0.69
ALB	**Cell number**								
	> 1 × 10^10^	52	50	Fixed	0	0.41	2.64	1.33 to 3.95	< 0.0001
	< 1 × 10^10^	119	160	Fixed	0	0.65	1.90	1.13 to 2.66	< 0.00001
	**Study sample size**								
	> 50	171	209	Fixed	0	0.55	2.09	1.44 to 2.75	< 0.00001
	≤ 50	57	58	Fixed	0	0.47	0.70	− 1.34 to 2.74	0.50
ALT	**Cell number**								
	> 1 × 10^10^	52	50	Random	69	0.07	− 10.67	− 28.02 to 6.67	0.23
	< 1 × 10^10^	99	140	Random	0	0.46	− 5.46	− 13.92 to 3.00	0.21
	**Study sample size**								
	> 50	171	209	Random	79	0.0007	− 8.08	− 17.02 to 0.87	0.08
	≤ 50	37	38	Random	60	0.11	− 10.45	− 34.83 to 13.93	0.40
PT	**Cell number**								
	> 1 × 10^10^	52	50	Random	90	0.002	− 2.84	− 6.07 to 0.39	0.08
	< 1 × 10^10^	39	77	Random			− 1.86	− 4.10 to 0.38	0.10
	**Study sample size**								
	> 50	37	38	Random	0	0.50	− 1.45	− 2.94 to 0.05	0.06
	≤ 50	111	146	Random	89	< 0.0001	− 2.40	− 4.98 to 0.19	0.07

*Abbreviations*: *Con group* control group (RT alone group), *Exp group* experimental group (RT plus ABMSC therapy), *RT* routing therapy, *TBIL* total bilirubin, *ALB* albumin, *ALT* alanine aminotransferase, *PT* prothrombin time, *RT* routing therapy, *ABMSC* autologous bone marrow stem cell

## Discussion

In recent years, ABMSC transplantation has been applied in several preclinical HBV-C studies [2, 19, 20]. ABMSC may act by promoting the survival and regeneration of functional hepatocytes and reducing collagen deposition to decelerate or halt cirrhosis progression [31–33]. Protocols in different trials show great diversity, which may be associated with different therapeutic effects, and no standardized protocol has been published to date. In this research, we performed a meta-analysis of a host of HBV-C clinical trials to systematically assess the effectiveness and safety of ABMSC transfusion administered via the hepatic artery.

In this meta-analysis, in comparison to HBV-C patients who received RT alone, those treated with ABMSC and RT combined therapy exhibited more favorable effects, including increased ALB and PTA levels, decreased TBIL, ALT, and AST levels, and a shortened PT. Liver fibrosis is one of the most important characteristics of cirrhosis [34]; its serological markers, including HA, LN, PC III, and CIV, indicated more significant relief of liver fibrosis after combined therapy. Moreover, both MELD and Child-Pugh scores were significantly lower in the combined therapy group than the RT alone group. Clinical symptoms of patients were markedly improved after ABMSC therapy, as demonstrated by improved appetite and relieved fatigue, abdominal distension, and ascitic fluid. These results indicated that the combination of ABMSC transplantation and RT had more satisfactory therapeutic effects for HBV-C patients than RT alone.

Safety is the top priority of a therapeutic strategy and a key factor for clinical application and further development. This analysis confirmed the safety of ABMSC transfusion in HBV-C treatment. The most common side effects during ABMSC therapy included fever, nausea, and vomiting, and no serious adverse events or death occurred during treatment.

Some factors may influence the therapeutic effects of ABMSC therapy and its evaluation. Our subgroup analysis indicated that the treatment effects might be associated with the dosage of injected cells, as well as by the sample size of the included trials. However, currently published studies probing the impact of these factors on the curative effects of ABMSC therapy have been insufficient, prompting further research and exploration.

There are some limitations of this analysis. First, the numbers of involved studies and patients were small, and the follow-up period was short. Second, the 10 included trials were all conducted in the Chinese population. ABMSC transfusion therapy has been used in many countries for liver diseases such as liver fibrosis, cirrhosis, and liver failure [18, 35–38]. Many trials conducted in other countries were excluded because of insufficient data, they were unrelated to HBV-C, or they involved the administration of ABMSC via a route other than the hepatic artery. Third, different trials evaluated treatment efficacy with different outcomes, so it was difficult to summarize the results using the same scale, leading to reduced statistical sample sizes. In addition, multiple factors, such as patient age and LC stage, might affect the therapeutic effect of ABMSC for HBV-C. However, based on the currently available literature, data are insufficient to perform a statistical analysis to evaluate such a correlation. We will continue to follow upcoming clinical trials to obtain relevant data when available. Finally, we noticed that there was a meta-analysis has been published during the process of submission [39], which may limit the novelty of this study to some extent. After a careful reading, we find that the focus between the two articles is different. (I) Some factors may have influence on the therapeutic effects of ABMSC therapy, such as ABMSC infusion methods (intravenous or hepatic artery infusion) and liver cirrhosis types (HBV- or HCV- or alcoholic-related or other types of cirrhosis). Our study predominantly focused on HBV-C patients treated by ABMSC transplantation via the hepatic artery, which can further eliminate the interference of other variable factors on ABMSC treatment. (II) Dynamic analysis of the treatment efficacy of ABMSC for HBV-C is necessary. In our analysis, biochemical (TBIL, ALB, ALT, and AST) and coagulation indicators (PT and PTA), MELD, and Child-Pugh scores were all evaluated between the two groups at baseline and weeks 2, 4, 8, 12, and 24 after therapy. (III) Many objective variables, such as hepatic fibrosis indicators and clinical symptoms, which related to therapeutic effect, were also evaluated in our study. In summary, we expect that our study will be valuable for the design of more comprehensive and controlled clinical trials.

## Conclusion

In summary, this meta-analysis illustrated that ABMSC transplantation via the hepatic artery combined with RT was safe and effective for the treatment of HBV-C. ABMSC transplantation showed outstanding benefits for HBV-C patients by improving their liver function and clinical symptoms. Therefore, ABMSC therapy is a promising treatment option for HBV-C patients.

## Supplementary information

**Additional file 1: Supplementary Figure 1.** Forest plot of the comparison of percentage of total bilirubin (TBIL, A), albumin (ALB, B), alanine aminotransferase (ALT, C), aspartate aminotransferase (AST, D) and prothrombin time (PT, E) between the experimental and control group before therapy. Control group, RT alone group; Experimental group, RT plus ABMSC therapy; ABMSC, autologous bone marrow stem cell; RT, routing therapy. The fixed-effects meta-analysis model (Inverse Variance method) was used.

**Additional file 2: Supplementary Figure 2.** Forest plot of the comparison of serum liver fibrosis markers including hyaluronic acid (HA, A), laminin (LN, B), type III procollagen (PC III, C) and type IV collagen (CIV, D) between the experimental and control group before therapy. Control group, RT alone group; Experimental group, RT plus ABMSC therapy; ABMSC, autologous bone marrow stem cell; RT, routing therapy. The fixed-effects meta-analysis model (Inverse Variance method) was used.

**Additional file 3: Supplementary Figure 3.** Forest plot of the comparison of albumin (excluding the study [20]) between the experimental and control group. Control group, RT alone group; Experimental group, RT plus ABMSC therapy; ABMSC, autologous bone marrow stem cell; RT, routing therapy. The fixed-effects meta-analysis model (Inverse Variance method) was used.

**Additional file 4: Supplementary Figure 4.** Forest plot of the comparison of alanine aminotransferase (excluding the study [20]) between the experimental and control group. Control group, RT alone group; Experimental group, RT plus ABMSC therapy; ABMSC, autologous bone marrow stem cell; RT, routing therapy. The random effects meta-analysis model (Inverse Variance method) was used.

**Additional file 5: Supplementary Figure 5.** Forest plot of the comparison of model for end-stage liver disease (excluding the study [20]) between the experimental and control group. Control group, RT alone group; Experimental group, RT plus ABMSC therapy; ABMSC, autologous bone marrow stem cell; RT, routing therapy. The fixed-effects meta-analysis model (Inverse Variance method) was used.

## Data Availability

Availability of data and materials can be assessed both in the “Material and methods” section and the “Results” section.

## Abbreviations

ABMSCs: Autologous bone marrow stem cells
ALB: Serum albumin
ALT: Alanine aminotransferase
AST: Aspartate aminotransferase
CHB: Chronic hepatitis B
CI: Confidence interval
CIV: Type IV collagen
CNKI: National Knowledge Infrastructure
HA: Hyaluronic acid
HBV: Hepatitis B virus
HBV-C: Hepatitis B virus-related cirrhosis
IFN-α: Interferon-α
LC: Liver cirrhosis
LN: Laminin
MD: Mean difference
MELD: Model for end-stage liver disease
NAs: Nucleotide analogs
OR: Odds ratio
PC III: Type III procollagen
PRISMA: Preferred Reporting Items for Systematic Reviews and Meta-Analyses
PT: Prothrombin time
PTA: Prothrombin activity
RT: Routine therapy
TBIL: Total bilirubin
VIP: Chinese Scientific Journal Database
