# Supplementary figures and images for: Autologous bone marrow stem cell transplantation via the hepatic artery for the treatment of hepatitis B virus-related cirrhosis: a PRISMA-compliant meta-analysis based on the Chinese population
Source: Stem Cell Res Ther. 2020 Mar 5;11:104. doi: 10.1186/s13287-020-01627-5 (PMC7059376; doi:10.1186/s13287-020-01627-5)

A

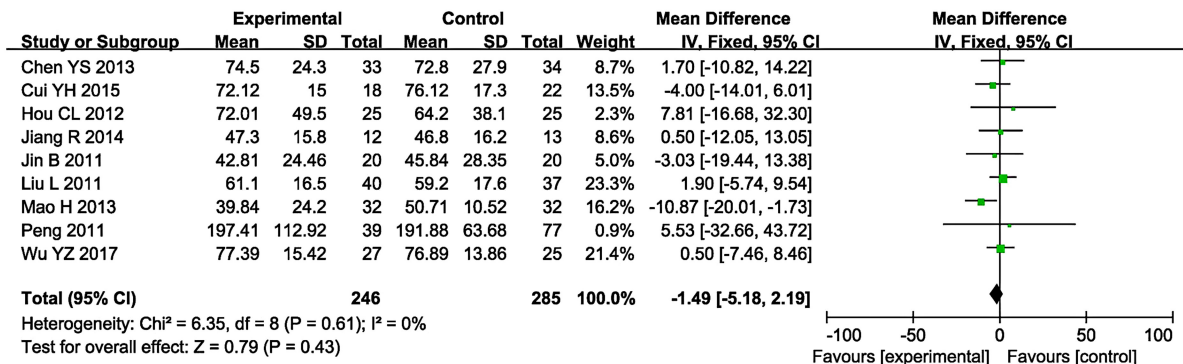

B

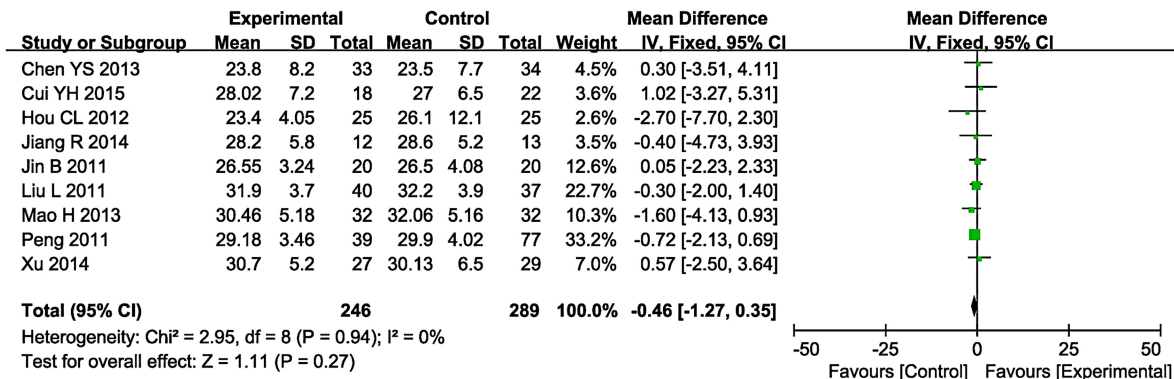

C

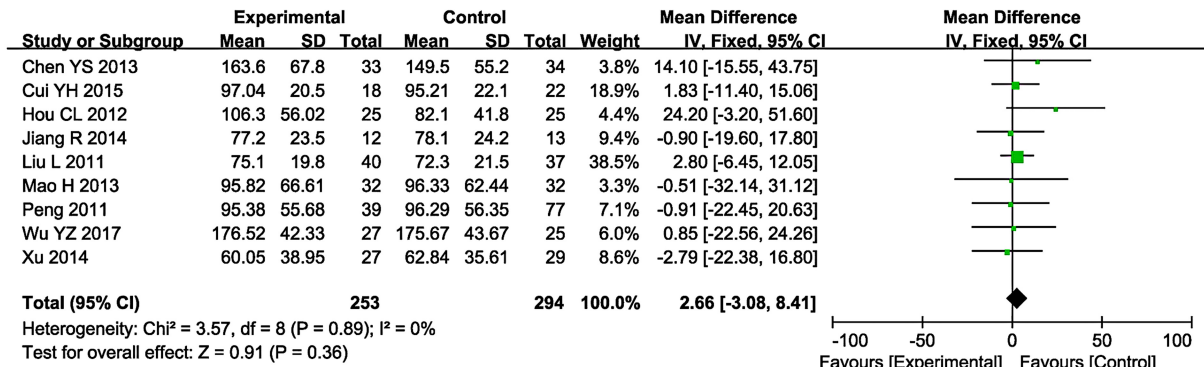

Supplement: Supplementary file 1 — Additional file 1: Supplementary Figure 1. Forest plot of the comparison of percentage of total bilirubin (TBIL, A), albumin (ALB, B), alanine aminotransferase (ALT, C), aspartate aminotransferase (AST, D) and prothrombin time (PT, E) between the experimental and control group before therapy. Control group, RT alone group; Experimental group, RT plus ABMSC therapy; ABMSC, autologous bone marrow stem cell; RT, routing therapy. The fixed-effects meta-analysis model (Inverse Variance method) was used. [file 13287_2020_1627_MOESM1_ESM.zip › Supplementary Figure 1-1.pdf]

D

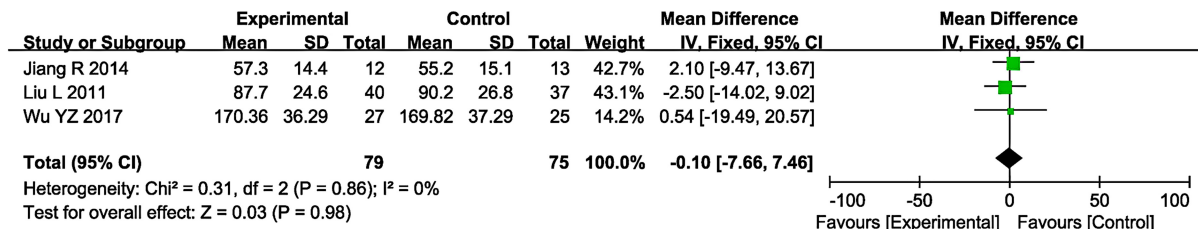

E

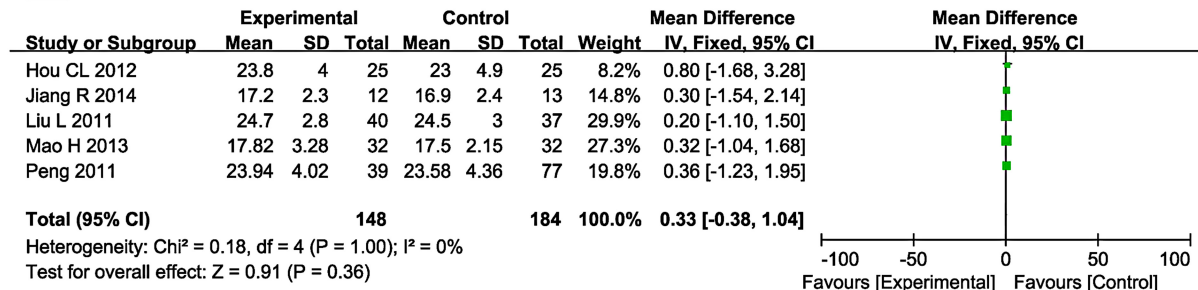

F

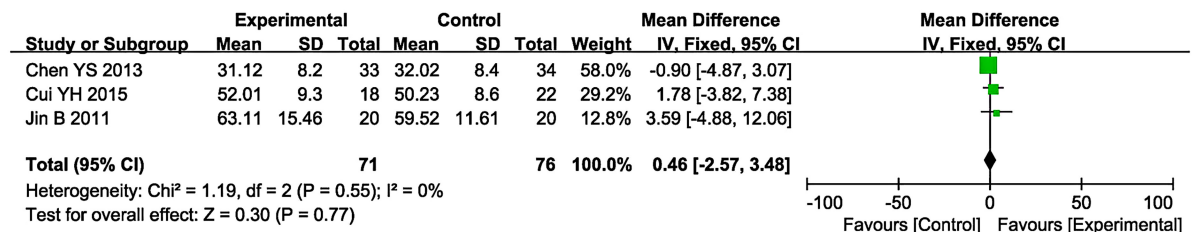

Supplement: Supplementary file 1 — Additional file 1: Supplementary Figure 1. Forest plot of the comparison of percentage of total bilirubin (TBIL, A), albumin (ALB, B), alanine aminotransferase (ALT, C), aspartate aminotransferase (AST, D) and prothrombin time (PT, E) between the experimental and control group before therapy. Control group, RT alone group; Experimental group, RT plus ABMSC therapy; ABMSC, autologous bone marrow stem cell; RT, routing therapy. The fixed-effects meta-analysis model (Inverse Variance method) was used. [file 13287_2020_1627_MOESM1_ESM.zip › Supplementary Figure 1-2.pdf]

A

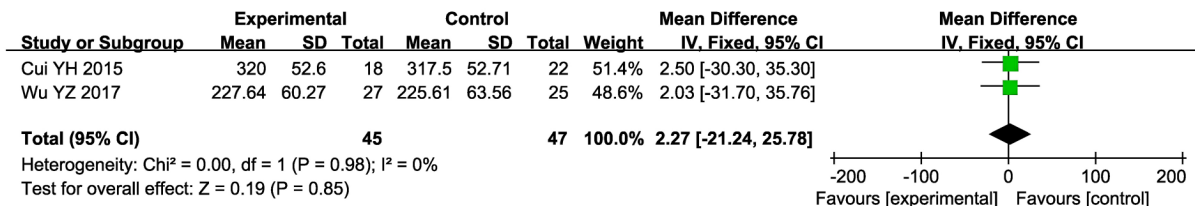

B

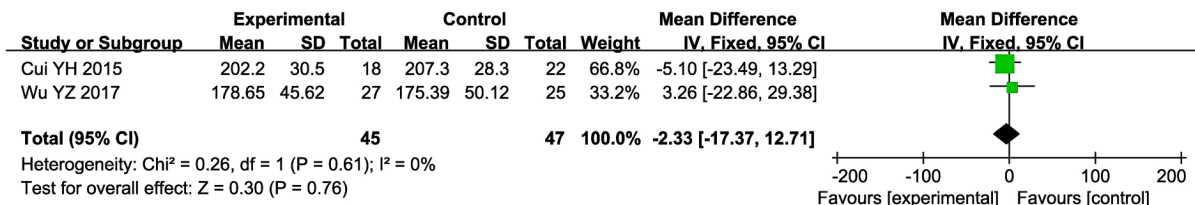

C

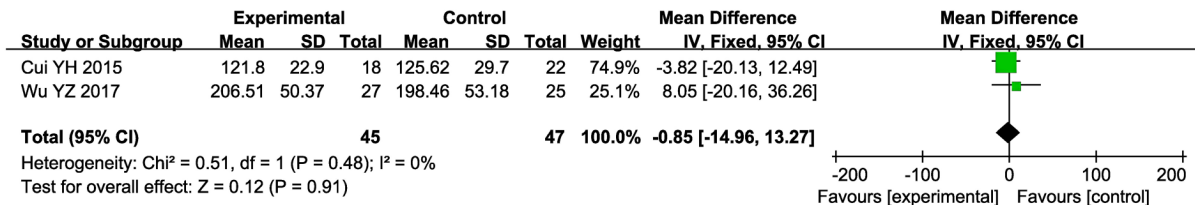

D

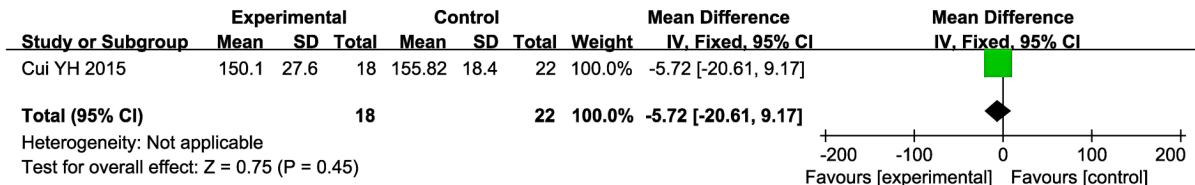

Supplement: Supplementary file 2 — Additional file 2: Supplementary Figure 2. Forest plot of the comparison of serum liver fibrosis markers including hyaluronic acid (HA, A), laminin (LN, B), type III procollagen (PC III, C) and type IV collagen (CIV, D) between the experimental and control group before therapy. Control group, RT alone group; Experimental group, RT plus ABMSC therapy; ABMSC, autologous bone marrow stem cell; RT, routing therapy. The fixed-effects meta-analysis model (Inverse Variance method) was used. [file 13287_2020_1627_MOESM2_ESM.pdf]

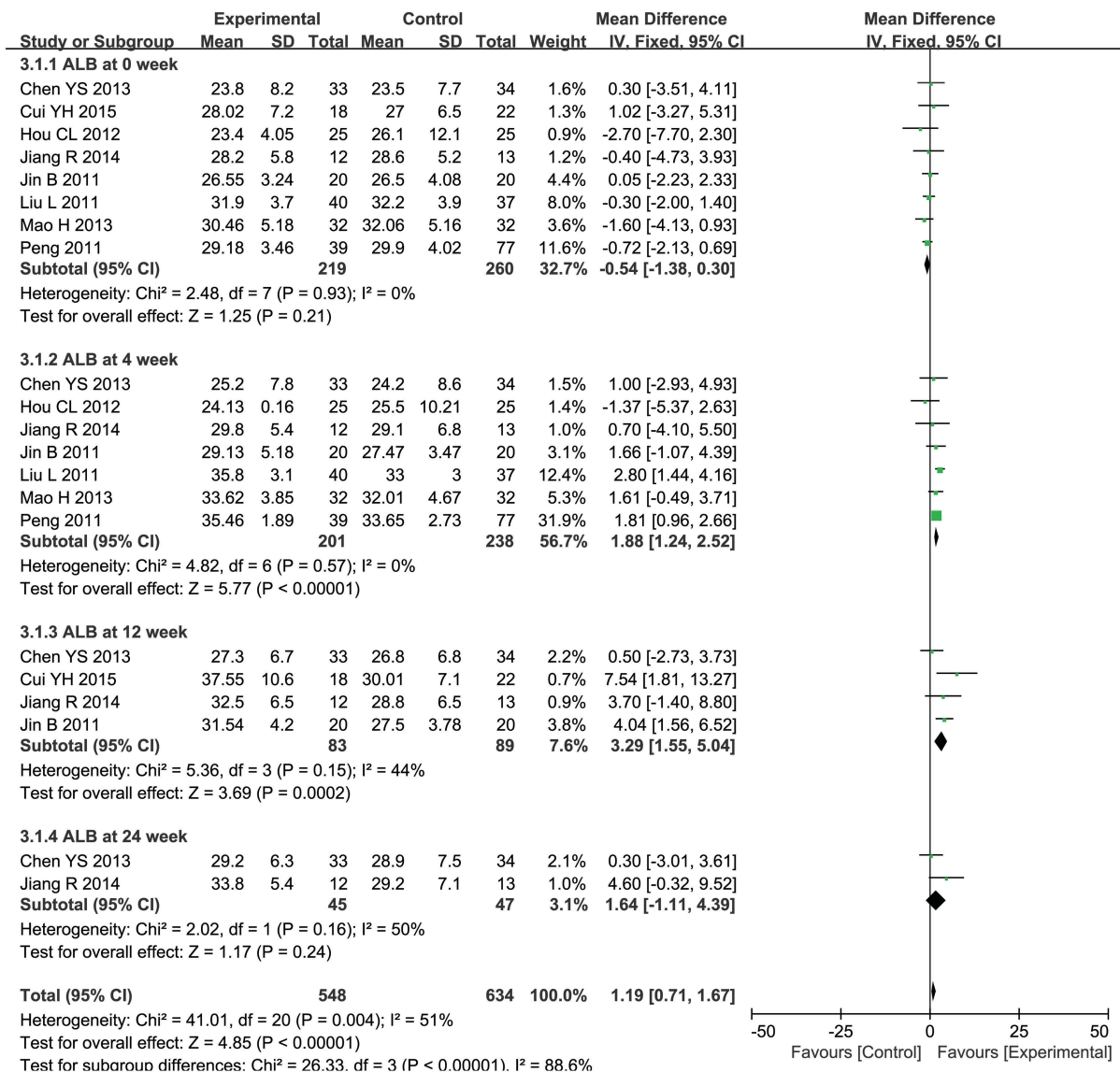

Supplement: Supplementary file 3 — Additional file 3: Supplementary Figure 3. Forest plot of the comparison of albumin (excluding the study [20]) between the experimental and control group. Control group, RT alone group; Experimental group, RT plus ABMSC therapy; ABMSC, autologous bone marrow stem cell; RT, routing therapy. The fixed-effects meta-analysis model (Inverse Variance method) was used. [file 13287_2020_1627_MOESM3_ESM.pdf]

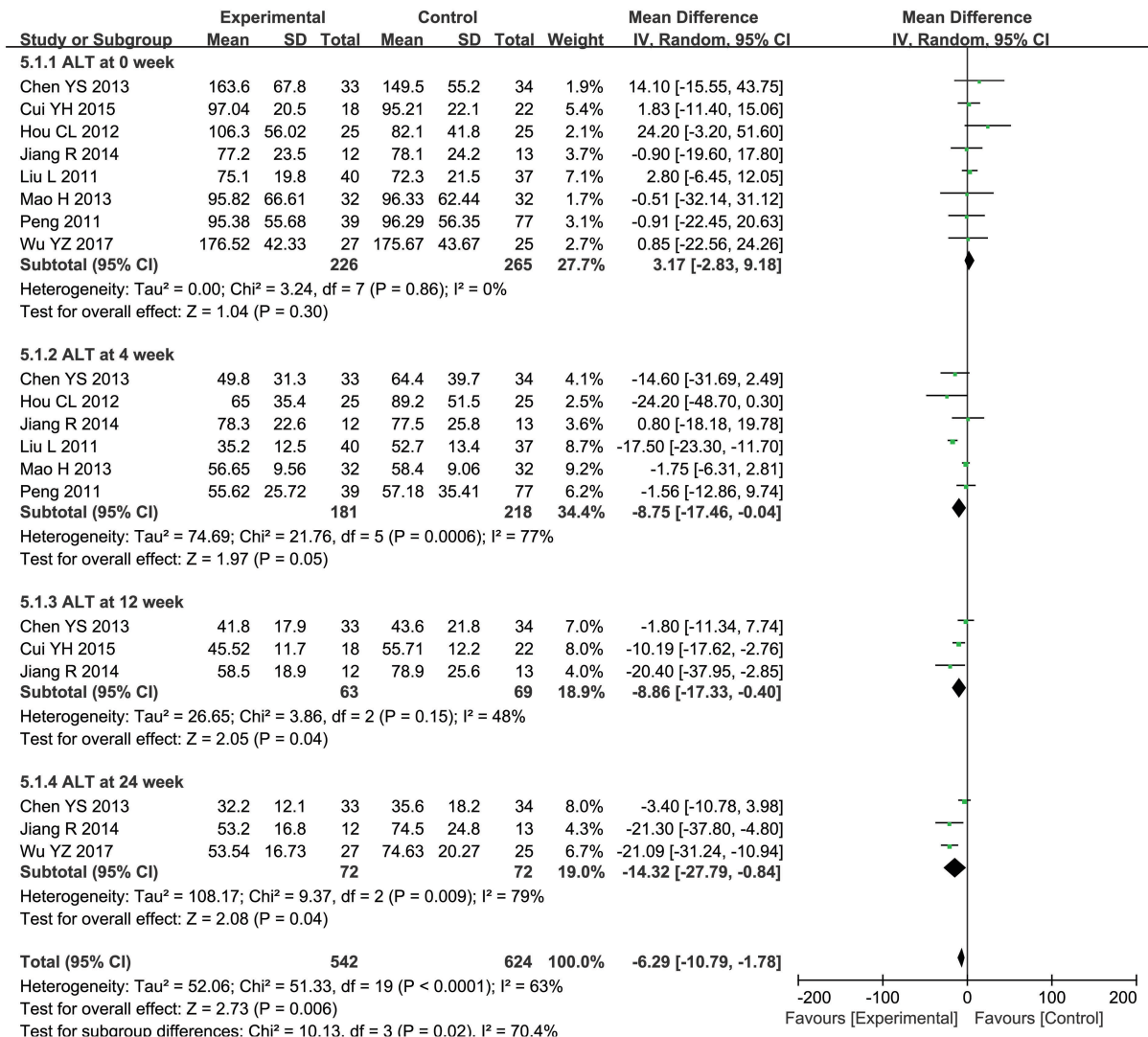

Supplement: Supplementary file 4 — Additional file 4: Supplementary Figure 4. Forest plot of the comparison of alanine aminotransferase (excluding the study [20]) between the experimental and control group. Control group, RT alone group; Experimental group, RT plus ABMSC therapy; ABMSC, autologous bone marrow stem cell; RT, routing therapy. The random effects meta-analysis model (Inverse Variance method) was used. [file 13287_2020_1627_MOESM4_ESM.pdf]

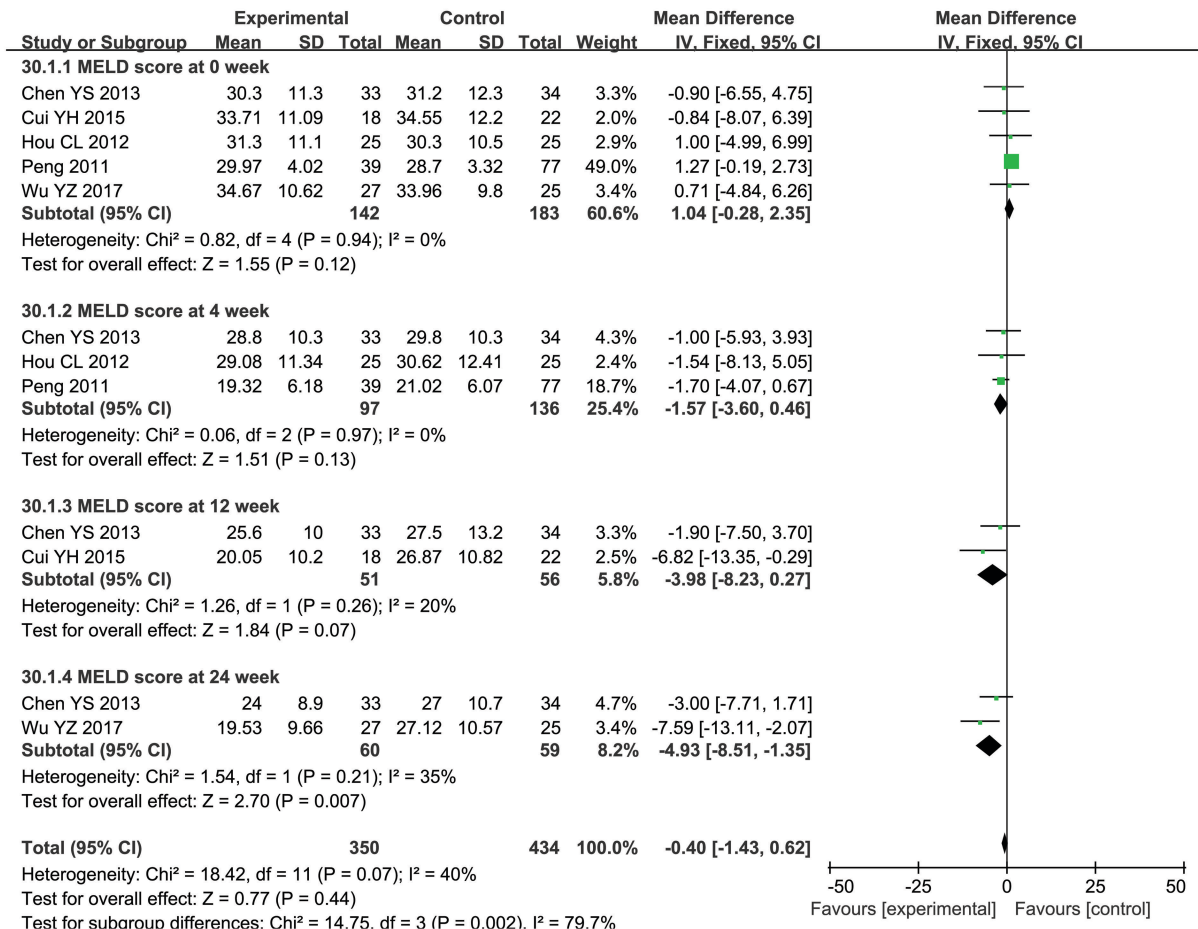

Supplement: Supplementary file 5 — Additional file 5: Supplementary Figure 5. Forest plot of the comparison of model for end-stage liver disease (excluding the study [20]) between the experimental and control group. Control group, RT alone group; Experimental group, RT plus ABMSC therapy; ABMSC, autologous bone marrow stem cell; RT, routing therapy. The fixed-effects meta-analysis model (Inverse Variance method) was used. [file 13287_2020_1627_MOESM5_ESM.pdf]
